# Areca nut components stimulate ADAM17, IL-1α, PGE2 and 8-isoprostane production in oral keratinocyte: role of reactive oxygen species, EGF and JAK signaling

**DOI:** 10.18632/oncotarget.7621

**Published:** 2016-02-23

**Authors:** Mei-Chi Chang, Chiu-Po Chan, Yi-Jane Chen, Hsiang-Chi Hsien, Ya-Ching Chang, Sin-Yuet Yeung, Po-Yuan Jeng, Ru-Hsiu Cheng, Liang-Jiunn Hahn, Jiiang-Huei Jeng

**Affiliations:** ^1^ Team of Biomedical Science, Chang-Gung University of Science and Technology, Kwei-Shan, Taoyuan City, Taiwan; ^2^ Department of Dentistry, Chang Gung Memorial Hospital, Taipei, Taiwan; ^3^ Laboratory of Pharmacology, Toxicology and Chemical Carcinogenesis, School of Dentistry and Department of Dentistry, National Taiwan University Hospital and National Taiwan University Medical College, Taipei, Taiwan; ^4^ Department of Dentistry, Mackay Memorial Hospial, and Mackay Junior College of Medicine, Nursing and Management, Taipei, Taiwan; ^5^ School of Dentistry, University of Cardenal Herrera, CEU, Valencia, Spain

**Keywords:** betel quid, oral cancer, epidermal growth factor, prostanoids, signal transduction

## Abstract

Betel quid (BQ) chewing is an etiologic factor of oral submucous fibrosis (OSF) and oral cancer. There are 600 million BQ chewers worldwide. The mechanisms for the toxic and inflammatory responses of BQ are unclear. In this study, both areca nut (AN) extract (ANE) and arecoline stimulated epidermal growth factor (EGF) and interleukin-1α (IL-1α) production of gingival keratinocytes (GKs), whereas only ANE can stimulate a disintegrin and metalloproteinase 17 (ADAM17), prostaglandin E_2_ (PGE_2_) and 8-isoprostane production. ANE-induced EGF production was inhibited by catalase. Addition of anti-EGF neutralizing antibody attenuated ANE-induced cyclooxygenase-2 (COX-2), mature ADAM9 expression and PGE_2_ and 8-isoprostane production. ANE-induced IL-1α production was inhibited by catalase, anti-EGF antibody, PD153035 (EGF receptor antagonist) and U0126 (MEK inhibitor) but not by α-naphthoflavone (cytochrome p450-1A1 inhibitor). ANE-induced ADAM17 production was inhibited by pp2 (Src inhibitor), U0126, α-naphthoflavone and aspirin. AG490 (JAK inhibitor) prevented ANE-stimulated ADAM17, IL-1α, PGE_2_ production, COX-2 expression, ADAM9 maturation, and the ANE-induced decline in keratin 5 and 14, but showed little effect on cdc2 expression and EGF production. Moreover, ANE-induced 8-isoprostane production by GKs was inhibited by catalase, anti-EGF antibody, AG490, pp2, U0126, α-naphthoflavone, Zinc protoporphyrin (ZnPP) and aspirin. These results indicate that AN components may involve in BQ-induced oral cancer by induction of reactive oxygen species, EGF/EGFR, IL-1α, ADAMs, JAK, Src, MEK/ERK, CYP1A1, and COX signaling pathways, and the aberration of cell cycle and differentiation. Various blockers against ROS, EGF, IL-1α, ADAM, JAK, Src, MEK, CYP1A1, and COX can be used for prevention or treatment of BQ chewing-related diseases.

## INTRODUCTION

Chewing betel quid (BQ) is popular in Taiwan, India and many Southeast Asian countries [1-3]. This habit increases the risk of oral leukoplakia, oral submucous fibrosis (OSF) and oral cancer. There are approximately 2-2.8 million BQ chewers in Taiwan [4] and 600 million BQ chewers worldwide [1]. BQ contains areca nut (AN), lime and inflorescence *Piper betle* with or without *Piper betle* leaf. However, the mechanisms and signaling transduction pathways of BQ chemical carcinogenesis are not clear. The induction of reactive oxygen species (ROS), damage to cellular targets (DNA, protein, lipid) after metabolic activation of BQ components by phase 1 enzymes (e.g., cytochrome P450s) [5], the cytotoxic effects of BQ constituents, keratinocyte inflammation and oncogene activation are suggested to be the contributing factors. ROS may be involved in the initiation, promotion and progression of cancer. During BQ chewing, ROS generation is confirmed by both *in vitro* [6, 7] and *in vivo* (in saliva) studies [8] and may induce oral squamous cell carcinoma (OSCC) in Papua New Guinea and other countries [2, 9], via auto-oxidation or metabolic activation by cytochrome p450 (CYP) enzymes [10]. The roles of ROS production by BQ components and the related upstream/downstream signaling in mediating cytotoxicity, aberrant differentiation and prostanoid production/tissue inflammation are crucial in BQ carcinogenesis.

Clinical studies have found the increased expression of a disintegrin and metalloproteinases (ADAMs) in OSCC of Taiwan and other country [11, 12]. Overexpression of epidermal growth factor (EGF) and EGF receptor (EGFR) is also noted in head and neck squamous cell carcinoma (HNSCC) [13]. EGFR can be activated by EGF, heparin-binding (HB)-EGF, transforming growth factor-α (TGF-α) and amphiregulin, as well as by ROS [14]. EGFR (HER1, erbB1) is a receptor tyrosine kinase (RTK) that modulates cell proliferation and differentiation via Janus kinase (JAK), Src and Ras/mitogen-activated protein kinases (MAPKs) signaling. Recently, the elevated expression of EGFR and MAPKs is crucial in the pathogenesis of oral cancer [15, 16]. Src is a non-receptor tyrosine kinase that may be activated by metals, ROS and ultraviolet (UV) irradiation [17]. Src kinase activity is necessary for EGF and other HER ligand signaling to signal transducer and activator of transcription (STAT) and MAPK pathways in various cancers [16-18].

ROS generated by toxicants can activate receptors, receptor-activated protein kinases and nuclear transcription factors, such as growth factor receptors, JAK, Src kinase, Ras signaling, MAPKs, the phosphoinositide-3-kinase (PI3K)/protein kinase B (Akt) pathway, and nuclear factor-κB (NF-κB) [15-17]. Recent studies have found the stimulation of various signal transduction pathways such as PI3K/Akt, NF-κB, mitogen-activated protein kinase kinase (MEK)/extracellular signal-regulated kinase (ERK), p38, c-jun N-terminal kinase (JNK), TGF-β/Smad and glycogen synthase kinase-3β (GSK-3β) by areca nut (AN) components in epithelial cells [19-21]. AN components also induce TGFβ/Smad and phospholipase C/inositol-triphosphate (IP3)/Ca^2+^/calmodulin, Rho, MEK/ERK and NF-κB signaling in oral fibroblasts [22-24]. During BQ chewing, ROS may be generated by auto-oxidation in saliva or via intracellular metabolic activation [1, 2]. Excessive ROS production by BQ components may lead to DNA/cell damage, inflammation, cell cycle regulation, apoptosis and gene expression with associated lipid peroxidation, protein modification and DNA damage. Interestingly, we found the activation of ROS, CYP1A1, EGFR, Ras, Src and hemeoxygenase-1 (HO-1) signaling by areca nut extract (ANE) to stimulate COX-2 expression/PGE_2_ production in gingival keratinocytes (GKs) [25]. BQ components further activated matrix metalloproteinase-2 (MMP-2) and MMP-9 in oral epithelial cells and cancer cells, contributing to the invasion and metastasis of OSCC [26, 27].

EGF/EGFR, tumor necrosis factor-α (TNF-α) and IL-1α may be involved in the sequential stages of carcinogenesis and tissue fibrosis. These effects occur via activation of receptors, ADAMs and TAK1 to cleave and release EGF [28]. An increased expression of cyclooxygenase-2 (COX-2) in different stages of oral cancer and marked inflammatory cell infiltration in OSF tissues may play a crucial role in the multi-step chemical carcinogenesis [29, 30]. Previous reports have found the induction of COX-2 and PGE_2_ production in GK by ANE via the activation of ROS, CYP1A1, EGFR, Ras, Src, (HO-1 and MEK/ERK [25, 31, 32]. It is intriguing to determine whether EGF, IL-1α, and ADAMs are activated by BQ components to induce the release of oxidative stress markers and inflammatory mediators—e.g., 8-isoprostane and PGE_2_ production—in oral mucosal cells. Moreover, signal transduction pathways such as ROS, JAK (a downstream molecule of EGFR), and MEK that mediate these cellular responses should be clarified. We hypothesized that BQ chewing may induce tissue inflammation, leading to OSF and oral cancer via stimulation of ROS, EGF/EGFR, JAK, IL-1α and ADAM17 (also called TNF-α converting enzyme, TACE) to impair differentiation and cell cycle progression, as well as the production of 8-isoprostane and PGE_2_ production in oral keratinocytes. These complex cross-talk events among EGF, EGFR, IL-1α, ADAM, JAK, Src and other signaling molecules may play an important role in BQ chewing-related diseases (e.g., cancer, OSF, and atherosclerosis). The results of this study may highlight our development of methods (small molecule inhibitors, antibodies etc.) for prevention and targeting therapy of BQ chewing-related diseases.

## RESULTS

### Effect of ANE and arecoline on EGF and IL-1α production by GKs

**Figure 1:**
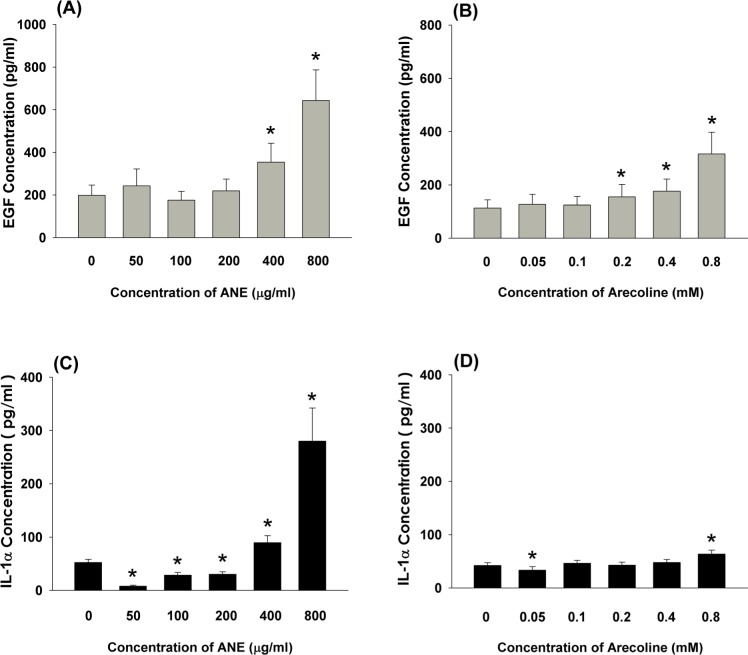
**A.** Stimulation of EGF level of GK by ANE (50-800 μg/ml) (n=27). **B.** Effect of arecoline on EGF level of GK (n=33), **C.** Stimulation of IL-1α production of GK by ANE (50-800 μg/ml) (n=14). **D.** Effect of arecoline on IL-1α production of GK (n=20). *denotes significant difference when compared with control (*P* < 0.05).

At concentrations of 400 and 800 μg/ml, ANE stimulated EGF secretion of GKs to 1.8 and 3.3-folds of control, respectively (Figure 1A). Interestingly, arecoline at concentrations of 0.2-0.8 mM also induced EGF secretion of GKs to 1.4-2.8 folds of control (Figure 1B). Similarly, ANE (400 and 800 μg/ml) induced IL-1α production of GKs by 1.7 to 5.4-folds of control, whereas ANE inhibited IL-1α production by GKs at concentrations of 50-200 μg/ml (Figure 1C). On the other hand, arecoline stimulated IL-1α production by GKs at a concentration of 0.8 mM, whereas it slightly inhibited IL-1α secretion by GKs at a concentration of 0.05 mM (Figure 1D).

### Effect of ANE and arecoline on ADAM17 (TACE) and 8-isoprostane production by GKs

**Figure 2:**
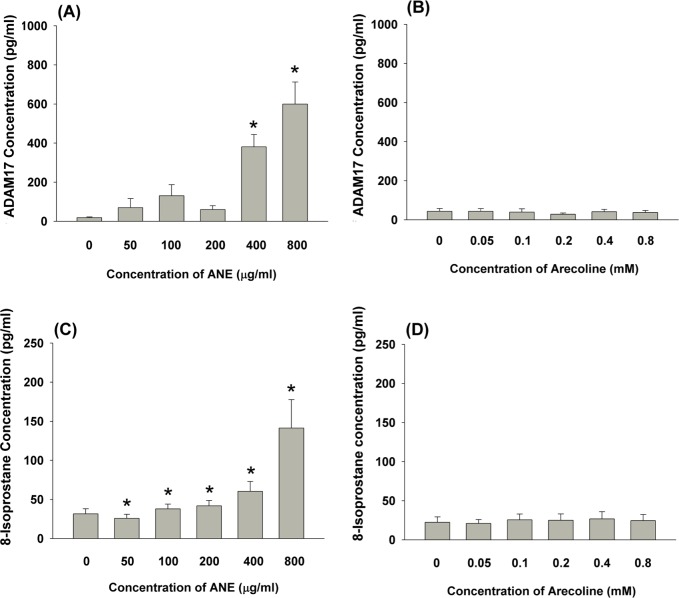
**A.** Stimulation of ADAM17 production of GK by ANE (50-800 μg/ml) (n=9). **B.** Effect of arecoline on ADAM17 production of GK (n=6), **C.** Stimulation of 8-isoprostane production of GK by ANE (50-800 μg/ml) (n=8). **D.** Effect of arecoline on 8-isoprostane production of GK (n=12). *denotes significant difference when compared with control (*P* < 0.05).

At concentrations of 400 and 800 μg/ml, ANE stimulated ADAM17 production of GKs by 16.5 and 21.9-folds relative to control (Figure 2A). Arecoline at all test concentrations (0.05-0.8 mM) showed little effect on ADAM17 production by GKs (Figure 2B). AN components have been shown to stimulate ROS production in various types of cells [1, 2]. Similarly, ANE (100-800 μg/ml) induced 8-isoprostane production of GKs by 1.2 to 4.5-folds of control, whereas ANE slightly inhibited 8-isoprostane production by GKs at a concentration of 50 μg/ml (Figure 2C). By contrast, arecoline showed little effect on 8-isoprostane production by GKs at concentrations of 0.05-0.8 mM (Figure 2D).

### Signaling for ANE-induced EGF production by GKs

**Figure 3:**
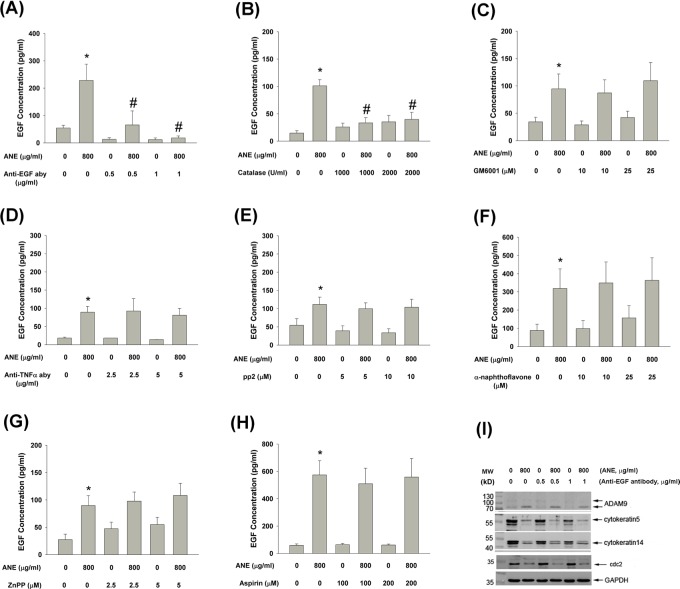
**A.** Pretreatment and co-incubation by anti-EGF neutralizing aby on ANE-induced EGF production in GK (n=7). **B.** Pretreatment and co-incubation by catalase on ANE-induced EGF production in GK (n=12). **C.** Pretreatment and co-incubation by GM6001 on ANE-induced EGF production in GK (n=5). **D.** Pretreatment and co-incubation by anti-TNFα neutralizing aby on ANE-induced EGF production in GK (n=3). **E.** Pretreatment and co-incubation by pp2 on ANE-induced EGF production in GK (n=6). **F.** Pretreatment and co-incubation by α-naphthoflavone on ANE-induced EGF production in GK (n=27). **G.** Pretreatment and co-incubation by ZnPP on ANE-induced EGF production in GK (n=18). **H.** Pretreatment and co-incubation by aspirin on ANE-induced EGF production in GK (n=10). *denotes significant difference when compared with solvent control. #denotes statistically significant difference when compared with ANE-treated group (*P* < 0.05). **I.** Effect of anti-EGF neutralizing aby on the ANE-induced alterations of ADAM9, keratin 5, keratin 14, cdc2 and GAPDH (control) protein expression as analyzed by western blotting. One representative western blot picture was shown.

To determine the upstream signaling molecules responsible for ANE-induced EGF production, we found that anti-EGF antibody (aby) effectively decreased the useful EGF content in the culture medium of GKs (Figure 3A). Pretreatment and co-incubation of catalase effectively prevented the ANE-induced EGF production by GKs (Figure 3B). On the other hand, GM6001 (an inhibitor of metalloproteinases), anti-TNFα neutralizing aby, pp2 (a Src inhibitor), α-naphthoflavone (a CYP1A1 inhibitor), Zinc proroporphyrin (ZnPP, a HO-1 inhibitor) and aspirin (a COX inhibitor) could not attenuate ANE-induced EGF production by GKs (Figure 3C, 3D, 3E, 3F, 3G, 3H). Anti-EGF aby attenuated the ANE-induced maturation of ADAM9 but showed little effect on the ANE-induced decline of cytokeratin 5, 14 and cdc2 expression (Figure 3I).

### Upstream signaling for ANE-induced IL-1α production by GKs

**Figure 4:**
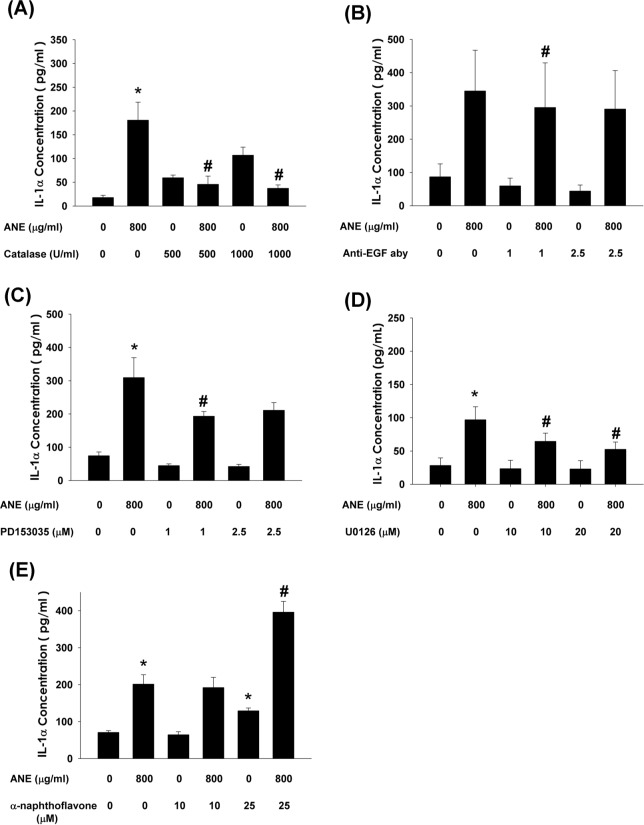
**A.** Pretreatment and co-incubation by catalase on ANE-induced IL-1α production in GK (n=11). **B.** Pretreatment and co-incubation by anti-EGF neutralizing aby on ANE-induced IL-1α production in GK (n=4). **C.** Pretreatment and co-incubation by PD153035 on ANE-induced IL-1α production in GK (n=11). **D.** Pretreatment and co-incubation by U0126 on ANE-induced IL-1α production in GK (n=8). **E.** Pretreatment and co-incubation by α-naphthoflavone on ANE-induced IL-1α production in GK (n=21). *denotes significant difference when compared with solvent control. #denotes statistically significant difference when compared with ANE-treated group (*P* < 0.05).

To identify the upstream signaling molecules responsible for ANE-induced IL-1α production, we found that catalase obviously suppressed ANE-induced IL-1α production by GKs (Figure 4A). Similarly, anti-EGF aby effectively decreased ANE-induced IL-1α production by GKs (Figure 4B). Pretreatment and co-incubation by PD153035 (an EGFR receptor antagonist) and U0126 (a MEK/ERK inhibitor) also effectively prevented ANE-induced EGF production by GKs (Figure 4C, 4D). By contrast, α-naphthoflavone enhanced ANE-induced IL-1α production by GKs (Figure 4E).

### Upstream signaling for ANE-induced ADAM17 production by GKs

**Figure 5:**
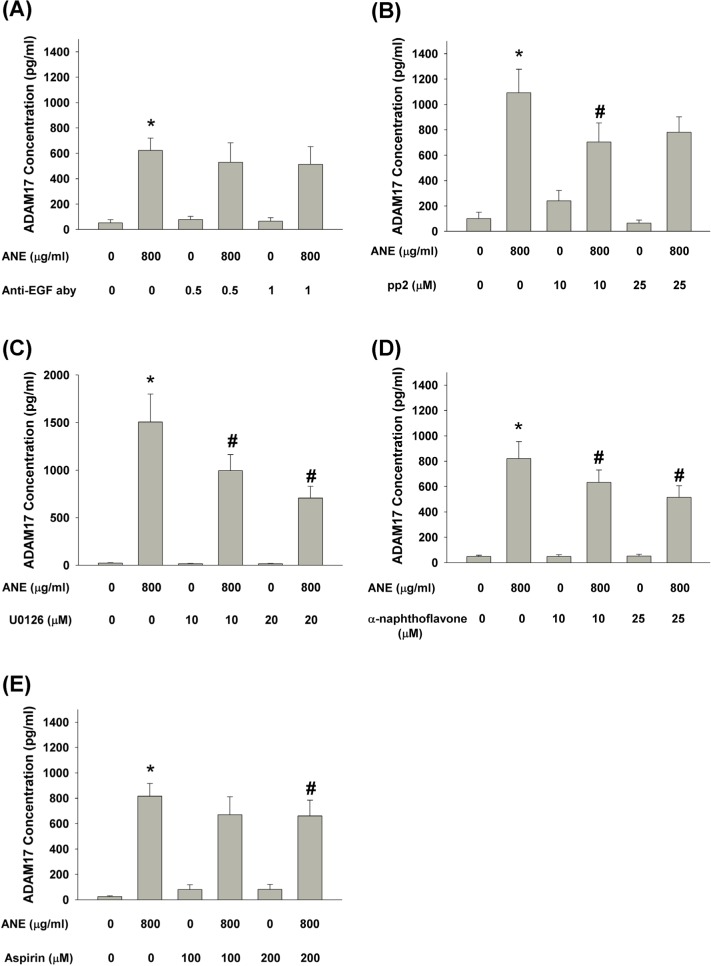
**A.** Pretreatment and co-incubation by anti-EGF neutralizing aby on ANE-induced ADAM17 production in GK (n=5). **B.** Pretreatment and co-incubation by pp2 on ANE-induced ADAM17 production in GK (n=4). **C.** Pretreatment and co-incubation by U0126 on ANE-induced ADAM17 production in GK (n=5). **D.** Pretreatment and co-incubation by α-naphthoflavone on ANE-induced ADAM17 production in GK (n=8). **E.** Pretreatment and co-incubation by aspirin on ANE-induced ADAM17 production in GK (n=10). *denotes significant difference when compared with solvent control. #denotes statistically significant difference when compared with ANE-treated group (*P* < 0.05).

To reveal the upstream signaling molecules responsible for ANE-induced ADAM17 production, we found that pretreatment and co-incubation by anti-EGF neutralizing aby slightly decreased ANE-induced ADAM17 production by GKs (*P* > 0.05) (Figure 5A). Pretreatment and co-incubation by pp2 and U0126 inhibited ANE-induced ADAM17 production by GKs (Figure 5B, 5C). Moreover, pretreatment and co-incubation by α-naphthoflavone and aspirin also attenuated ANE-induced ADAM17 production by GKs (Figure 5D, 5E).

### Role of JAK signaling in ANE-induced effects on GKs

**Figure 6:**
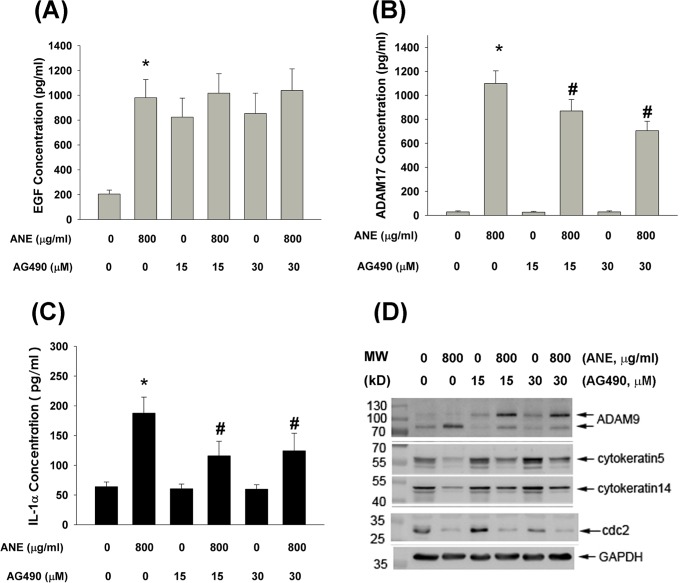
**A.** Pretreatment and co-incubation by AG490 (15 and 30 μM, a JAK inhibitor) on ANE-induced EGF production in GK (n=21). **B.** Pretreatment and co-incubation by AG490 on ANE-induced ADAM17 production in GK (n=5). **C.** Pretreatment and co-incubation by AG490 on ANE-induced IL-1α production in GK (n=47). *denotes significant difference when compared with solvent control. #denotes statistically significant difference when compared with ANE-treated group (*P* < 0.05). **D.** Effect of AG490 on ANE-induced alterations of ADAM9, keratin 5, keratin 14, cdc2 and GAPDH (control) protein expression as analyzed by western blotting. One representative western blot picture was shown.

Because JAKs are important signaling molecules responsible for EGFR-mediated events, we further tested and found that AG490 (a JAK inhibitor) could not prevent ANE-induced EGF production by GKs (Figure 6A). By contrast, AG490 attenuated ANE-induced ADAM17 and IL-1α production by GKs (Figure 6B, 6C). Accordingly, ANE inhibited keratin 5, keratin 14, cdc2 protein expression, whereas ANE stimulated the protein expression of mature ADAM9 (84 KD) but had no marked effect on precursor ADAM9 (105 KD). (Figure 6D). AG490 may prevent the inhibitory effect of ANE on keratin 5 and keratin 14. Additionally, AG490 suppressed the stimulatory effect of ANE on the protein expression of mature ADAM9, with an increase in precursor ADAM9 expression (Figure 6D).

### Role of EGF and JAK on ANE-induced COX-2 expression and PGE2 production by GKs

**Figure 7:**
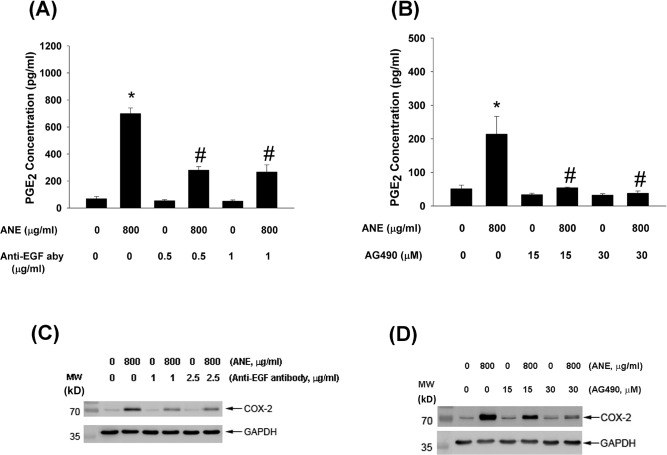
**A.** Pretreatment and co-incubation by anti-EGF neutralizing aby (0.5 and 1 μg/ml) on ANE-induced PGE_2_ production in GK. **B.** Pretreatment and co-incubation by AG490 (15 and 30 μM) on ANE-induced PGE2 production in GK. Results were expressed as Mean ± SE. *denotes significant difference when compared with solvent control. #denotes statistically significant difference when compared with ANE-treated group (*P* < 0.05). **C.** Pretreatment and co-incubation by anti-EGF neutralizing aby on ANE-induced COX-2 protein expression of GK. **D.** Pretreatment and co-incubation by AG490 on ANE-induced COX-2 protein expression of GK. One representative western blotting picture was shown.

To understand the role of EGF and JAK in mediating ANE-induced COX-2 expression and PGE_2_ production, anti-EGF aby and AG490 were used to suppress the effect of EGF/EGFR and JAK signaling. Interestingly anti-EGF aby effectively inhibited ANE-induced PGE_2_ production and COX-2 expression in GKs (Figure 7A, 7C). Similarly, AG490 also markedly suppressed ANE-induced PGE_2_ production and COX-2 expression in GKs (Figure 7B, 7D).

### Effect of catalase, anti-EGF antibody, IL-1 receptor associated kinase (IRAK) inhibitor, AG490, pp2, U0126, α-naphthoflavone, ZnPP and aspirin on ANE-induced 8-isoprostane production by GKs

**Figure 8:**
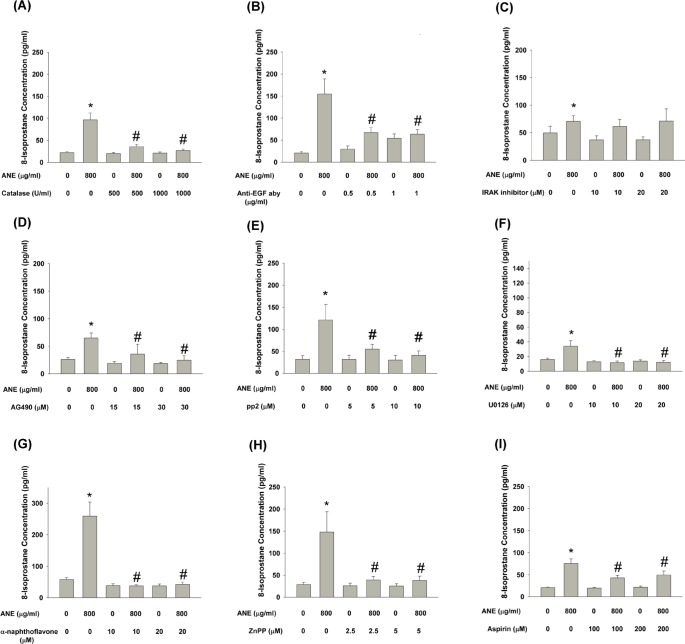
**A.** Pretreatment and co-incubation by catalase on ANE-induced 8-isoprostane production in GK. **B.** Pretreatment and co-incubation by anti-EGF neutralizing aby on ANE-induced 8-isoprostane production in GK. **C.** Pretreatment and co-incubation by IRAK inhibitor on ANE-induced 8-isoprostane production in GK. **D.** Pretreatment and co-incubation by AG490 on ANE-induced 8-isoprostane production in GK. **E.** Pretreatment and co-incubation by pp2 on ANE-induced 8-isoprostane production in GK. **F.** Pretreatment and co-incubation by U0126 on ANE-induced 8-isoprostane production in GK. **G.** Pretreatment and co-incubation by α-naphthoflavone on ANE-induced 8-isoprostane production in GK. **H.** Pretreatment and co-incubation by ZnPP on ANE-induced 8-isoprostane production in GK. **I.** Pretreatment and co-incubation by aspirin on ANE-induced 8-isoprostane production in GK. *denotes significant difference when compared with solvent control. #denotes statistically significant difference when compared with ANE-treated group (*P* < 0.05).

Generally, 8-isoprostane is considered an oxidative stress marker and product. In this study, catalase effectively prevented ANE-induced 8-isoprostane production by GKs (Figure 8A). We further tested whether the induction of EGF by ANE is important for this event. Anti-EGF neutralizing aby evidently attenuated the ANE-induced 8-isoprostane production (Figure 8B). However, IRAK inhibitor (inhibitor of IL-1) showed little preventive effects on ANE-induced 8-isoprostane production (Figure 8C). To elucidate the role of JAK (a downstream molecule of EGF/EGFR) signaling, AG490 pretreatment and co-incubation almost completely inhibited ANE-induced 8-isoprostane production by GKs (Figure 8D). Similar inhibitory effects of pp2 (Figure 8E) and U0126 (Figure 8F) on ANE-induced 8-isoprostane production were also noted. Moreover, to clarify the role of various metabolic enzymes in 8-isoprostane production, α-naphthoflavone could attenuate ANE-induced 8-isoprostane production by GKs (Figure 8G). Consistently, ZnPP and aspirin could also prevent ANE-induced 8-isoprostane production by GKs (Figure 8H, 8I).

Under these study conditions, the inhibitors anti-EGF aby, anti-TNFα aby, GM6001, IRAK inhibitor, PD153035, AG490, pp2, U0126, α-naphthoflavone and aspirin showed no marked influence on ANE-induced cytotoxicity of GKs as analyzed by the MTT assay (data not shown). Catalase showed protection against ANE cytotoxicity, whereas ZnPP enhanced ANE cytotoxicity [25].

## DISCUSSION

BQ chewing increases the risk of oral cancer and OSF, where oral mucosal inflammation is frequently noted [1-3]. Exogenous carcinogens may induce tumor promotion and progression by stimulating tissue inflammation through the induction of inflammatory mediators release from localized epithelial cells, fibroblasts and other tissue cells [33-36]. Accordingly, BQ components stimulate the production of various inflammatory mediators such as PGE_2_, PGF_2α_, IL-6, and TNFα in different cell types [1, 25, 31, 32, 37]. Previous studies have observed the stimulation of many signal transduction pathways, such as PI3K/Akt, NF-κB, MEK/ERK, p38, JNK, TGF-β/Smad and GSK-3β pathways, by AN components in epithelial cells [19-21]. ANE further activates ROS, CYP1A1, EGFR, Ras, Src and HO-1 signaling to stimulate COX-2 expression/PGE_2_ production in GKs [25]. To further elucidate the upstream signaling and downstream effective molecules, we found that AN components induce EGF, IL-1α and ADAM17 secretion by GKs. This finding may partly explain why the elevation of EGF, IL-1α, and ADAM17 expression is frequently observed in clinical OSCC and HNSCC [11-13, 38]. Accordingly, arecoline induces TGF-β2, HO-1, and IL-1α expression in HaCaT epithelial cells via ROS/p38 signaling [39]. EGF promotes the proliferation, invasion and epithelial mesenchymal transition of oral cancer cells [40, 41]. On the other hand, IL-1α alters immune status, stimulates cytokines production and the proliferation of cancer-associated fibroblasts and promotes cancer progression [38, 42]. Moreover, ADAM17 stimulates the invasion, stemness and tumorigenesis of HNSCC by cleavage of CD44 [43, 44]. These results suggest that BQ components are involved in oral carcinogenesis via the induction of EGF, IL-1α and ADAM17.

Recently, we have found the stimulation of EGFR phosphorylation and activation by ANE [25], possibly due to the induction of EGF production by ANE as found in this study. This event is inhibited by catalase, but not by GM6001, anti-TNFα aby, pp2, α-naphthoflavone, ZnPP and aspirin, suggesting that ANE-induced EGF production is correlated to ROS, but not by TNFα production, proteinase cleavage, Src, CYP1A1, HO-1 and COX. EGF can be an early response signaling molecule for ANE-induced cellular events in GKs. Moreover, anti-EGF aby attenuates ANE-induced ADAM9 maturation, but not the ANE-induced decline of cytokeratin 5, 14 and cdc2, indicating the presence of differential signaling pathways responsible for different downstream effective molecules. Anti-EGF aby and AG490 suppress the ANE-induced COX-2 expression, PGE_2_ and 8-isoprostane production, but not cdc2 expression of GK. During BQ chewing, ROS may be generated by auto-oxidation of BQ components in saliva or via their intracellular metabolic activation [1, 2]. BQ-induced ROS overproduction is correlated to DNA/cell damage, tissue inflammation, cell cycle regulation, apoptosis and gene expression with associated lipid peroxidation, protein modification and DNA damage. Recently, we have found the activation of ROS, CYP1A1, EGFR, Ras, Src and HO-1 signaling by ANE to induce COX-2 expression/PGE_2_ production in GK [25]. Moreover, EGF can activate EGFR to stimulate cell proliferation, differentiation, invasion and metastasis via stimulation of downstream JAK, Src, Ras/MAPKs and PI3K/Akt signaling [14, 16-18]. GW2974, a dual inhibitor of EGFR and ErbB2 tyrosine kinase, may attenuate the 7,12-dimethylbenz[a]anthracene (DMBA)-induced hamster cheek pouch tumor with concomitant reduction of tissue PGE_2_, indicating the presence of crosstalk between EGFR and arachidonic acid metabolism [45]. Studies also reveal the upregulation of COX-2 and EGFR in oral leukoplakia and oral carcinogenesis [46]. In this study, ROS-EGF/EGFR- and JAKs-COX-2 signaling pathways are shown to contribute to oral mucosal inflammation and carcinogenesis in BQ chewers. ANE-induced ADAM9 maturation and decrease of cytokeratin expression are correlated to JAK. ANE has been shown to PI3K/Akt, EGFR and COX signaling and contribute to BQ carcinogenesis [25, 47, 48]. However, additional signaling molecules are present to down-regulate cdc2 by ANE.

ROS are critical molecules for stimulation of ANE-induced PGE_2_ production in GK [25, 31]. To know more about the role of ROS in BQ carcinogenesis, we interestingly found that ROS is necessary for the ANE-induced EGF, IL-1α, and 8-isoprostane production. However, ANE at lower concentrations partly inhibited the IL-1α and 8-isoprostane production, possibly because ANE also contains some anti-oxidative components. IL-1α is involved in tissue inflammation, immune modulation and carcinogenesis via binding to IL-1 receptor to trigger signal transduction pathways such as IL-1 receptor (IL-1R)-associated kinase (IRAK) and TGFβ-activated kinase-1 (TAK1) [49, 50]. 8-Isoprostane has been used as a disease marker for obesity, ischemia-reperfusion injury, and cancer [51]. It may activate thromboxane receptors in response to oxidative injury [52]. Exposure to ANE may stimulate ROS and thereby downstream signaling pathways such as EGF/EGFR, IL-1α/IL-1R and 8-isoprostane/receptor to stimulate oral carcinogenesis. This may explain why ROS may activate receptors, receptor-activated protein kinases and nuclear transcription factors, including growth factor receptors, JAK, Src kinase, Ras signaling, MAPKs, PI3K/Akt pathway, NF-kB [16-18]. In addition to catalase, the ANE-induced IL-1α production is prevented by anti-EGF aby, PD153035 and U0126, but enhanced by α-naphthoflavone. These results suggest that ANE-induced IL-1α production of GK is mediated by ROS, EGF/EGFR and MEK/ERK activation. Similarly IL-1α production and nuclear localization are correlated to ROS levels, EGFR activation and MEK/ERK in fibrosarcoma, skin keratinocytes and in cerebral ischemia injury [53, 54]. Furthermore, IL-1α and TNF-α are important mediators involved in carcinogenesis and fibrosis of many organs via activation of receptor activation/TAK1 signaling [55, 56]. GK expressed various types of CYP enzymes mainly CYP1A1, 2C8/19, 2E1, and 3A3/3A4, and may involve in ANE-induced COX-2 expression and PGE_2_ production in GK [25]. Interestingly α–naphthoflavone by itself stimulates IL-1α production. This may partly explain the inhibition of CYP1A1/CYP1A2 by α–naphthoflavone enhanced the ANE-induced IL-1α production. The involvement of CYP1A1/CYP1A2 and its inhibition by α-naphthoflavone on ANE-induced events suggest that possibly metabolic activation of ANE components is necessary for some of the ANE-induced carcinogenic events [25] and increase the risk of OSF and oral cancer [57, 58].

Since EGFR ligands can be shed from plasma membrane by metalloproteinases and sheddases - a disintegrin and metalloproteinases (ADAMs). ADAM10, 12, 17 are the major sheddases of EGFR ligands in response to stimuli such as G-protein coupled receptors, growth factors, cytokines, wounding and phorbol ester etc. [59]. Over-expression of ADAMs (ADAM9, 10, 12, and 17 etc.) is popularly noted in epithelial inflammation and carcinogenesis [60] and increased expression of certain ADAMs may enhance tumor cells invasion, proliferation *in vitro* and promote tumor formation *in vivo*. ADAM17 may enhance the invasion of oral cancer [43]. An increased expression of ADAM10 is found in OSCC of Taiwan [11] and expression ADAM17 in head/neck SCC in Germany [12]. MMP2 and MMP-9 also contribute to BQ-related oral carcinogenesis by promotion of cancer invasion and metastasis [26, 27]. In this study, we further found the stimulation of ADAM9 maturation and ADAM17 secretion by ANE, suggesting the involvement of ADAM9 and ADAM17 in BQ carcinogenesis. ANE-induced ADAM17 secretion can be suppressed by pp2, U0126, α-naphthoflavone and aspirin, indicating this event is associated with Src, MEK/ERK, CYP1A1 and COX signaling. Src is a non-receptor tyrosine kinase that is activated by metals, ROS and UV irradiation [17]. Src overexpression has been found in head/neck cancers. Activated Src may induce downstream signaling of MAPKs, NF-kB and PI3K. Moreover, BQ components can stimulate Src activation and ERK to promote cancer cells' migration and motility [61].

Previous studies show the association between tissue inflammation and cancer/fibrosis with an elevation of COX-2 expression and prostanoid production in oral cancer and precancer [30]. AN components may induce tissue injury and inflammation, COX-2 expression and PGE_2_ production in GK via ROS, EGFR, Src, and MEK/ERK signaling [25, 31, 32]. In this study, ANE is further found to induce 8-isoprostane production. PGE_2_ is involved in oral carcinogenesis by induction of sustaining epithelial hyperplasia, angiogenesis, immuno-suppression and tumor metastasis. The 8-isoprostane has been suggested as an oxidative stress marker during chemical carcinogenesis and may induce vasoconstriction but inhibit angiogenesis [62-64]. 8-Isoprostane levels in serum, urine and exhaled breath condensate are used as the disease marker in tissue fibrosis, prostate and lung cancer [65-67], suggesting the potential use of 8-isoprostane as a marker of oral cancer and OSF. ANE-induced PGE_2_ production is related to ROS, EGFR, Src, MEK/ERK, CYP1A1 and HO-1 [25], and in addition, EGF and JAK signaling in this study. Interestingly, ANE-induced 8-isoprostane production of GK is prevented by catalase, anti-EGF aby, IRAK inhibitor, AG490, pp2, U0126, α-naphthoflavone, ZnPP and aspirin. These results demonstrate that ANE-induced 8-isoprostane production in GK is related to ROS, EGF and IL-1 production and the downstream signaling via IRAK, JAK, Src, MEK/ERK. CYP1A1, HO-1 and aspirin are also associated with these processes.

**Figure 9:**
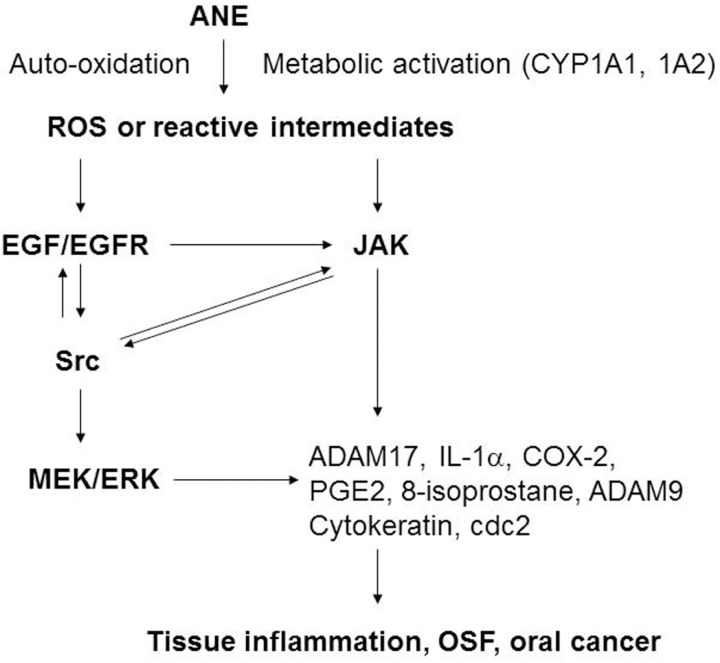
The signaling mechanism of ANE-induced molecular changes (ADAM17, IL-1α, COX-2, PGE_2_, 8-isoprostane, cytokeratins etc.) in gingival keratinocytes (GK) The possible signal transduction pathways (ROS, EGF/EGFR, Src, JAK, MEK/ERK, CYP1A1 etc.) responsible for the ANE-induced changes in cell proliferation, differentiation and inflammation of GK were shown.

Based on this study and other prior reports [1, 2, 6, 25, 32], we conclude that AN components play crucial roles in the pathogenesis of BQ-induced oral cancer and OSF possibly via induction of ROS, EGF/EGFR, JAK, Src, MEK/ERK, IL-1α, ADAMs, CYP1A1, HO-1 and COX signaling pathways, as well as the aberration in cell cycle- and differentiation-related proteins of oral keratinocytes (Figure 9). Auto-oxidation or metabolic activation of ANE components by CYP1A1 may generate ROS and reactive intermediates. ROS may induce multiple signaling pathways such as EGF/EGFR, JAK, Src, IL-1α/IL-1R, HO-1 to regulate COX-2, 8-isoprostane, keratin 5, keratin 14 and cdc2 expression. BQ induced impairment of growth, differentiation and inflammation may be involved in the pathogenesis of OSF and OSCC. Future development and application of various antioxidants, small molecule inhibitors, targeting antibodies and other novel methods against ROS, EGF, IL-1α, ADAM, JAK, Src, MEK, CYP1A1, and COX signal transduction pathways can be used for prevention or treatment of BQ chewing-related cancer and other diseases in the future.

## MATERIALS AND METHODS

### Materials

Keratinocyte growth medium (KGM-SFM), pituitary gland extract and EGF were purchased from Gibco (Life Technologies, BRL, Grand Island, NY, USA). Arecoline, catalase, aspirin and 3-(4,5-dimethyl-thiazol-2-yl)-2,5-diphenyl-tetrazolium bromide (MTT) were obtained from Sigma (Sigma Chemical Company, St. Louis, MO, USA). ANE was prepared and weighed as previously described [25, 32]. PGE_2_ and 8-isoprostane (8-iso-PGF_2α_) Enzyme-linked immunosorbant assay (ELISA) kits were obtained from Cayman Chemical Company (Ann Arbor, MI, USA). Human IL-1α ELISA kits were from PeproTech (Rocky Hill, NJ, USA), whereas ADAM17 and EGF ELISA kits were from R&D Systems (Minneapolis, MN, USA). GM6001, PD153035, AG490, pp2, U0126, α-naphthoflavone and ZnPP were obtained from Tocris or Cayman. Anti-EGF, anti-IL-1α and anti-TNFα neutralizing aby were obtained from PeproTech. Antibodies against COX-2, cdc2, ADAM9 and glyceraldehyde-3-phosphate dehydrogenase (GAPDH) were from Santa Cruz, whereas antibodies for cytokeratin 5 and cytokeratin 14 were from GeneTex (GeneTex International Corporation, Global, Hsin-Chu, Taiwan). IRAK-1/4 inhibitor was from APExBIO (Houston, TX, USA).

### Culture of gingival keratinocytes (GKs)

GKs were cultured as described previously [25, 32]. With the approval of the Ethics Committee of National Taiwan University Hospital, human gingiva (with a gingivitis index < 1) was obtained during clinical crown-lengthening procedures with proper written informed consent by the patients. Most of the subepithelial connective tissue of the gingiva was first removed using a surgical knife, and then tissues were cut into small pieces, placed onto culture dishes and cultured in KGM-SFM with supplements. The cell passages of GKs ranging from 1 to 3 were used through this study.

### Effect of ANE and arecoline on 8-isoprostane, EGF, IL-1α, and ADAM17 production by GKs

Near-confluent GKs in 6-well culture plates were exposed to 2 ml of fresh medium containing various concentrations of ANE and arecoline. Cells were further incubated for 24 h. Culture medium was collected for the analysis of 8-isoprostane, EGF, IL-1α, and ADAM17 levels by ELISA.

### Effect of catalase, anti-EGF aby, AG490, pp2, anti-TNFα aby, anti-IL-1α aby, GM6001, U0126, α-naphthoflavone, ZnPP and aspirin on ANE-induced EGF, IL-1α, ADAM17, production by GK and concomitant cytotoxicity

Near-confluent GKs in 6-well culture plates were exposed to 2 ml of fresh medium containing catalase, AG490, pp2, anti-EGF aby, anti-IL-1α aby, anti-TNFα aby, GM6001, U0126, α-naphthoflavone, ZnPP and aspirin for 30 min, and then ANE was added. Cells were further incubated for 24 h. Culture medium was collected for the analysis of EGF, IL-1α, and ADAM17 levels by ELISA. Cytotoxicity was evaluated by the MTT assay as described previously [25, 32].

### Effect of catalase, anti-EGF aby, AG490, pp2, anti-TNF-α aby, anti-IL-1α aby, GM6001, U0126, α-naphthoflavone, ZnPP and aspirin on ANE-induced 8-isoprostane and PGE_2_ production by GKs

Near-confluent GKs in 6-well culture plates were exposed to 2 ml of fresh medium containing catalase, AG490, pp2, anti-EGF aby, anti-IL-1α aby, anti-TNFα aby, GM6001, U0126, α-naphthoflavone, ZnPP and aspirin for 30 min, and then ANE was added. Cells were further incubated for 24 h. Culture medium was collected for the analysis of 8-isoprostane and PGE_2_ level by ELISA.

### Effect of ANE on ADAM9, cdc2, cytokeratin 5, cytokeratin 14, COX-2 protein expression and its modulation by anti-EGF aby and AG490 by GKs

Near-confluent GKs were exposed to ANE with or without pretreatment and co-incubation by anti-EGF aby or AG490 for 24 hours. Cells were washed with PBS, disrupted in lysis buffer (10 mm Tris-HCl, pH 7; 140 mm sodium chloride; 3 mm magnesium chloride; 0.5% NP-40; 2 mm phenylmethylsulfonyl fluoride; 1% aprotinin; and 5 mm dithiothreitol). The concentrations of proteins were measured using Bio-Rad protein assay kits. Equal amounts of protein (30-50 μg/lane) were then loaded for 12% SDS-polyacrylamide gel electrophoresis (Scie-Plas, UK) and then transferred to polyvinylidene fluoride (PVDF) membranes. The membranes were blocked for 30 min at room temperature in a blocking agent (20 mM Tris, pH 7.4; 125 mM NaCl; 0.2% Tween 20; 5% nonfat dry milk; and 0.1% sodium azide) and incubated with anti-human ADAM9, cdc2, cytokeratin 5, cytokeratin 14, COX-2 and GAPDH antibodies for 2 hours. The membranes were then washed 3 times with tris-buffered saline with Tween-20 (TBST: 10 mM Tris, pH 7.5; 100 mM NaCl, 0.1% Tween-20 for 10 min each, and finally incubated with secondary aby for 1 h [25, 32]. The membranes were rinsed 4 times with TBST, and then ECL reagents were added and the immuno-reactive bands were eventually developed by ECL reagent and visualized/photographed using an Image Reader (LAS-4000; Fujifilm, Japan).

### Statistical analysis

Four or more separate experiments were performed. The results were expressed as the mean ± SE and analyzed by paired Student's t-test. A *P* value < 0.05 was considered to indicate a statistically significant difference between 2 study groups.

## References

[1] IARC Betel-quid and areca-nut chewing and some areca-nut derived nitrosamines (2004). IARC Working Group on the Evaluation of Carcinogenic Risks to Humans. IARC Monogr Eval Carcinog Risks Hum.

[2] Jeng JH, Chang MC, Hahn LJ (2001). Role of areca nut in betel quid-associated chemical carcinogenesis: current awareness and future perspectives. Oral Oncol.

[3] Warnakulasuriya S, Trivedy C, Peters TJ (2002). Areca nut use: an independent risk factor for oral cancer. Br Med J.

[4] Ko YC, Chiang TA, Chang SJ, Hsieh SF (1992). Prevalence of betel quid chewing habit in Taiwan and related sociodemographic factors. J Oral Pathol Med.

[5] Sundqvist K, Liu Y, Nair J, Bartsch H, Arvidson K, Graftrom RC (1989). Cytotoxic and genotoxic effects of areca nut-related compounds in cutured human buccal epithelial cells. Cancer Res.

[6] Nair UJ, Friesen M, Nair J, Bussachini V, Friesen M, Bartsch H (1987). Formation of reactive oxygen species and 8-OH dG in DNA in vitro with betel quid ingredients. Chem Biol Interact.

[7] Liu TY, Chen CL, Chi CW (1996). oxidative damage to DNA induced by areca nut extract. Mutat Res.

[8] Nair UJ, Nair J, Friesen MD, Bartsch H, Ohshima H (1995). Ortho- and meta-tyrosine formation from phenylalanine in human saliva as a marker of hydroxyl radical generation during betel quid chewing. Carcinogenesis.

[9] Thomas SJ, MacLennan R (1992). Slaked lime and betel nut cancer in Papua New Guinea. Lancet.

[10] Nemeikaite-Ceniene A, Imbrasaite A, Sergediene E, Cenas N (2005). Quantitative structure-activity relationships in prooxidant cytotoxicity of polyphenols: role of potential of phenoxyl radical/phenol redox couple. Arch Biochem Biophys.

[11] Ko SY, Lin SC, Wong YK, Liu CJ, Chang KW, Liu TY (2007). Increase of disintegrin metalloprotease 10 (ADAM10) expression in oral squamous cell carcinoma. Cancer Lett.

[12] Kornfeld JW, Meder S, Wohlberg M, Friedrich RE, Rau T, Riethdorf L, Loning T, Pantel K, Riethdorf S (2011). Overexpression of TACE and TIMP-3 mRNA in head and neck cancer: association with tumour development and progression. Br J Cancer.

[13] Kim S, Grandis JR, Rinaldo A, Takes RP, Ferlito A (2008). Emerging perspectives in epidermal growth factor receptor targeting in head and neck cancer. Head & Neck.

[14] El-Abaseri TB, Putta S, Hansen LA (2006). Ultraviolet irradiation induces keratinocyte proliferation and epidermal hyperplasia through the activation of the epidermal growth factor receptor. Carcinogenesis.

[15] Chiang WF, Liu SY, Yen CY, Lin CN, Chen YC, Lin SC, Chang KW (2008). Association of epidermal growth factor receptor (EGFR) gene copy number amplification with neck lynph node metastasis in areca-associated oral carcinomas. Oral Oncol.

[16] Murugan AK, Munirajan AK, Tsuchida N (2012). Ras oncogenes in oral cancer: the past 20 years. Oral Oncol.

[17] Leonard SS, Harris GK, Shi XL (2004). Redox-active metal ions, reactive oxygen species, and apoptosis. Free Radic Biol Med.

[18] Silva CM (2004). Role of STATs as downstream signal transducers in Src family kinase-mediated tumorigenesis. Oncogene.

[19] Lin SC, Lu SY, Lee SY, Lin CY, Chen CH, Chang KW (2005). Areca (betel) nut extract activates mitogen-activated protein kinases and NF-kappaB in oral keratinocytes. Int J Cancer.

[20] Dai JP, Chen XX, Zhu DX, Wan QY, Chen C, Wang GF, Li WZ, Li KS (2014). Panax notoginseng saponins inhibit areca nut extract-induced oral submucous fibrosis in vitrol. J Oral Pathol Med.

[21] Ji WT, Lee CI, Chen JY, Cheng YP, Yang SR, Chen JH, Chen HR (2013). Areca nut extract induces pyknotic necrosis in serum-starved oral cells via increasing reactive oxygen species and inhibiting GSK3β: an implication of cytopathic effects in betel quid chewers. PLoS ONE.

[22] Chang MC, Lin LD, Wu HL, Ho YS, Hsien HC, Wang TM, Jeng PY, Cheng RH, Hahn LJ, Jeng JH (2013). Areca nut-induced buccal fibroblast contraction and its signaling: a potential role in oral submucous fibrosis – a precancer condition. Carcinogenesis.

[23] Khan I, Kumar N, Pant I, Narra S, Kondaiah P (2012). Activatino of TGF-pathway by areca nut constituents: a possible cause of oral submucous fibrosis. PLoS One.

[24] Yu CC, Tsai CH, Hsu HI, Chang YC (2013). Elevation of S1004 expression in buccal mucosal fibroblasts by arecoline: involvement in the pathogenesis of oral submucous fibrosis. PLoS One.

[25] Chang MC, Chen YJ, Chang HH, Chan CP, Yeh CY, Wang YL, Cheng RH, Hahn LJ, Jeng JH (2014). Areca nut components affect COX-2, cyclin B1/cdc25C and keratin expression, PGE2 production in keratinocyte is related to reactive oxygen species, CYP1A1, Src, EGFR and Ras signaling. PLoS ONE.

[26] Liu YC, Lin MH, Liu SY, Chiang WF, Chen LL, Chen TC, Cheng YC, Hsu KC, Cheng PC, Lee CH, Liu YC (2010). Possible mechanism of betel-quid-extract-induced expression of metalloproteinase-2. J Formos Med Assoc.

[27] Chang MC, Chan CP, Wang WT, Chang BE, Lee JJ, Tseng SK, Yeung SY, Hahn LJ, Jeng JH (2013). Toxicity of areca nut ingredients: activation of Chk1/Chk2, inducing cell cycle arrest, and regulation of MMP-9 and TIMPs production in SAS epithelial cells. Head & Neck.

[28] Colomiere M, Ward AC, Riley C, Trenerry MK, Cameron-Smith D, Findlay J, Ackland L, Ahmed N (2009). Cross talk of signals between EGFR and IL-6R through JAK2/Stat3 mediate epithelial-mesenchymal transition in ovarian carcinomas. Br J Cancer.

[29] Marks F, Furstenberger G (2000). Cancer chemoprevention through interruption of multistage carcinogenesis. The lessons learnt by comparing mouse skin carcinogenesis and human large bowel cancer. Eur J Cancer.

[30] Pandey M, Prakash O, Santhi WS, Soumithran CS, Pillai RM (2008). Overexpression of COX-2 gene in oral cancer is independent of stage of disease and degree of differentiation. Int J Oral Maxillofac Surg.

[31] Jeng JH, Ho YS, Chan CP, Wang YJ, Hahn LJ, Lei D, Hsu CC, Chang MC (2000). Areca nut extract upregulates prostaglandin production, cyclooxygenase-2 mRNA and protein expression of human oral keratinocytes. Carcinogenesis.

[32] Chang MC, Wu HL, Lee JJ, Lee PH, Chang HH, Hahn LJ, Lin BR, Chen YJ, Jeng JH (2004). The induction of prostaglandin E2 production, interleukin6 production, cell cycle arrest and cytotoxicity in primary oral keratinocytes and KB cancer cells by areca nut ingredients is differentially regulated by MEK/ERK activation. J Biol Chem.

[33] Parsonnet J (1997). Molecular mechanisms for inflammation-promoted carcinogenesis of cancer - The sixteenth International Symposium of Sapporo Cancer Seminar. Cancer Res.

[34] Barker JN, Mitra RS, Griffiths CE, Dixit VM, Nickoloff BJ (1991). Keratinocytes as initiators of inflammation. Lancet.

[35] Hogaboam CM, Steinhauser ML, Chensue SW, Kunkel SL (1998). Novel roles for chemokines and fibroblasts in interstitial fibrosis. Kidney Int.

[36] Weitzman SA, Gordon LI (1990). Inflammation and cancer: Role of phagocyte-generated oxidants in carcinogenesis. Blood.

[37] Jeng JH, Wang YJ, Chiang BL, Lee PH, Chan CP, Ho YS, Wang TM, Lee JJ, Hahn LJ, Chang MC (2003). Roles of keratinocyte inflammation in oral cancer: regulating the prostaglandin E2, interleukin-6 and TNF-alpha production of oral epithelial cells by areca nut extract and arecoline. Carcinogenesis.

[38] Woods KV, El-Naggar A, Clayman GL, Grimm EA (1998). Variable expression of cytokines in human head and neck squamous cell carcinoma cell lines and consistent expression in surgical specimens. Cancer Res.

[39] Thangjam GS, Kondaiah P (2009). Regulation of oxidative-stress responsive genes by arecoline in human keratinocytes. J Periodont Res.

[40] Richter P, Umbreit C, Franz M, Berndt A, Grimm S, Uecker A, Bohmer FD, Kosmehl H, Berndt A (2011). EGF/TGFβ1 co-stimulation of oral squamous cell carcinoma cells causes an epithelial-mesenchymal transition cell phenotype expressing laminin 332. J Oral Pathol Med.

[41] Zhang X, Jung IH, Hwang YS (2015). EGF enhances low-invasive cancer cell invasion by promoting IMP-3 expression. Tumour Biol.

[42] Bae JY, Kim EK, Yang DH, Zhang X, Park YJ, Lee DY, Che CM, Kim J (2014). Reciprocal interaction between carcinoma-associated fibroblasts and squamous carcinoma cells through interleukin-1α induces cancer progression. Neoplasma.

[43] Takamune Y, Ikebe T, Nagano O, Shinohara M (2008). Involvement of NF-kappaB-mediated maturation of ADAM-17 in the invasion of oral squamous cell carcinoma. Biochem Biophys Res Commun.

[44] Kamarajan P, Shin JM, Qian Z, Matte B, Zhu J, Kapila YL (2013). ADAM17-mediated CD44 cleavage promotes orasphere formation or stemness and tumorigenesis in HNSCC. Cancer Med.

[45] Sun Z, Sood S, Li N, Yang PY, Newman RA, Yang CS, Chen XX (2008). Chemoprevention of 7,12-dimethylbenz[a]-anthracene (DMBA)-induced oral carcinogenesis in hamster cheek pouch by topical application of a dual inhibitor of epidermal growth factor receptor (EGFR) and ErbB2 tyrosine kinases. Oral Oncol.

[46] Lippman SM, Sudbo J, Hong WK (2005). Oral cancer prevention and the evolution of molecular-targeted drug development. J Clin Oncol.

[47] Sundqvist K, Graftstrom RC (1992). Effects of areca nut on growth, differentiation and formation of DNA damage in cultured buccal epithelial cells. Int J Cancer.

[48] Tseng YH, Chang CS, Liu TY, Kao SY, Chang KW, Lin SC (2007). Areca nut extract treatment down-regulates involucrin in normal human oral keratinocyte through PI3K/AKT activation. Oral Oncol.

[49] Jain A, Kaczanowska S, Davila E (2014). IL-1 receptor-associated kinase signaling and its role in inflammation, cancer progression and therapy resistance. Front Immunol.

[50] Gabay C, Lamacchia C, Palmer G (2010). IL-1 pathways in inflammation and human diseases. Nat Rev Rheumatol.

[51] Milne GL, Dai Q, Roberts LJ (2015). The isoprostanes – 25 years later. Biochim Biophys Acta.

[52] Morrow JD (2006). The isoprostanes – unique products of arachidonate peroxidation: their role as mediators of oxidant stress. Curr Pharm Des.

[53] McCarthy DA, Ranganathan A, Subbaram S, Flaherty NL, Patel N, Trebak M, Hempel N, Melendez JA (2013). Redox-control of the alarmin, interleukin-1α. Redox Biol.

[54] Hobbs RM, Watt FM (2003). Regulation of interleukin-1alpha expression by integrins and epidermal growth factor receptor in keratinocytes from a mouse model of inflammatory skin disease. J Biol Chem.

[55] Berasain C, Perugorria MJ, Latasa MU, Castilo J, Goni S, Santamaria M, Prieto J, Avila MA (2009). The epidermal growth factor receptor: a link between inflammation and liver cancer. Exp Biol Med.

[56] Ma FY, Tesch GH, Ozols E, Xie M, Schneider MD (2011). Nikolic-Paterson DJ TGF-1-activated kinase-1 regulates inflammation and fibrosis in the obstructed kidney. Am J Physiol Renal Physiol.

[57] Li N, Hu Q, Jiang C, Hu Y, Yuan Y, Jian X, Tang Z, Guo F (2011). Novel genetic biomarkers for susceptibility to oral submuous fibrosis: cytochrome P450 3A. Med Hypothesis.

[58] Topcu Z, Chiba I, Fujieda M, Shibata T, Ariyoshi N, Yamazaki H, Sevgican F, Muthumala M, Kobayashi H, Kamataki T (2002). CYP2A6 gene deletion reduces oral cancer risk in betel quid chewers in Sri Lanka. Carcinogenesis.

[59] Higashiyama S, Nanba D (2005). ADAM-mediated ectodomain shedding of HB-EGF in receptor cross-talk. Biochim iophys Acta.

[60] Cesaro A, Abakar-Mahamat A, Brest P, Lassalle S, Selva E, Filippi J, Hebuterne X, Hugot JP, Doglio A, Galland F, Naquet P, Vouret-Craviari V, Mograbi B, Hofman PM (2009). Differential expression and regulation of ADAM17 and TIMP-3 in acute inflamed intestinal epithelia. Am J Physiol Gastrointest Liver Physiol.

[61] Chiu CC, Chen BH, Hour TC, Ciang WF, Wu YJ, Chen CY, Chen HR, Chan PT, Liu SY, Chen JY (2010). Betel quid extract promotes oral cancer cell migration by activating a muscarinic M4 receptor-mediated signaling cascade involving SFKs and ERK1/2. Biochem Biophys Res Commun.

[62] Hoffman SW, Moore S, Ellis EF (1997). Isoprostanes: free radical-generated prostaglandins with constrictor effects on cerebral arterioles. Stroke.

[63] Bobrowska B, Tokarz A, Bialek S, Seweryn M (2011). Effect of dietary supplementation on the prognostic valu of urinary and serum 8-isoprostaglandin F2alpha in chemically-induced mammary carcinogenesis in the rat. Lipids Health Dis.

[64] Benndorf RA, Schwedhelm E, Gnann A, Taheri R, Kom G, Didie M, Steenpass A, Ergun S, Boger RH (2008). Isoprostanes inhibit vascular endothelial growth factor-induced endothelial cell migration, tube formation, and cardiac vessel sprouting in vitro, as well as angiogenesis in vivo via activation of the thromboxane A2 receptor: a potential link between oxidative stress and impaired angiogenesis. Circ Res.

[65] Malli F, Bardaka F, Tsilioni I, Karetsi E, Gourgoulianis KI, Daniil Z (2013). 8-isoprostane levels in serum and bronchoalveolar lavage in idiopathic pulmonary fibrosis and sarcoidosis. Food Chem Toxicol.

[66] Brys M, Morel A, Forma E, Krzeslak A, Wilkosz J, Rozanski W, Olas B (2013). Relationship of urinary isoprostanes to prostate cancer occurrence. Mol Cell Biochem.

[67] Stathopoulos D, Loukides S, Syrigos K (2014). 8-Isoprostane in exhaled breath condensate of patients with non-small cell lung cancer: the effect of chemotherapy. Anticancer Res.

